# Piezoelectric amino acids and peptides: Mechanisms, molecular engineering, and biomedical applications

**DOI:** 10.1016/j.isci.2026.117396

**Published:** 2026-09-07

**Authors:** Mengyang Zhang, Hao Wang, Han Liu, Dezhi Zhao, Jiawei Wu, Tengzhi Ma

**Affiliations:** 1 Xi’an Key Laboratory of Innovative Drug Research for Heart Failure, Faculty of Life Sciences and Medicine, Northwest University, 229 Taibai North Road, Xi’an 710069, China

**Keywords:** Bioengineering, Electrical engineering, Materials science

## Abstract

Piezoelectric amino acids and peptides have attracted growing attention in biomedical research because of their intrinsic piezoelectricity, superior biocompatibility, and biodegradability. The electrical signals generated by these materials resemble endogenous bioelectric signals in tissues, making them promising for biosensing, tissue regeneration, antimicrobial therapy, and cancer treatment. This review summarizes the piezoelectric mechanisms, classifications, molecular modification approaches, external-field-assisted fabrication methods, and reported biomedical applications of amino acid- and peptide-based piezoelectric biomaterials. It also outlines key challenges and future directions for biodegradable bioelectronic systems. By integrating these aspects, this review provides a theoretical foundation for developing targeted piezoelectric amino acid and peptide materials with excellent piezoelectric performance, biocompatibility, and biodegradability, and for promoting their clinical application.

## Graphical abstract

**Figure:**
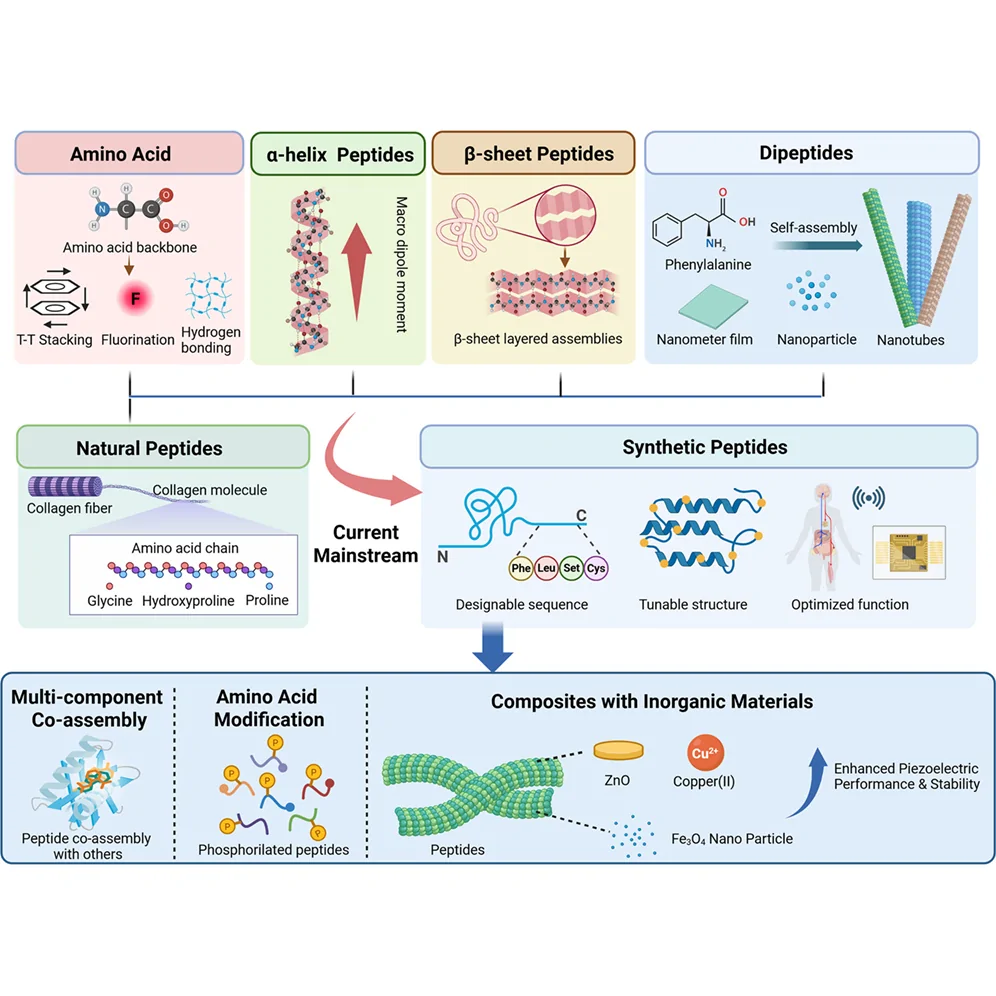

## Introduction

The piezoelectric effect describes the electromechanical coupling in non-centrosymmetric materials, whereby mechanical deformation induces electrical polarization and vice versa.^1^^,^^2^^,^^3^ In biological systems, endogenous electrical signals generated by mechanoelectric conversion play critical roles in tissue development, regeneration, and cellular communication.^4^ Consequently, piezoelectric biomaterials have emerged as promising candidates in fields including self-powered bioelectronics, tissue regeneration, and implantable sensors.^5^^,^^6^^,^^7^

Conventional piezoelectric materials primarily comprise inorganic piezoelectric ceramics and organic piezoelectric materials. Inorganic materials such as lead zirconate titanate (PZT) and barium titanate exhibit outstanding piezoelectric performance; however, they suffer from inherent brittleness, poor mechanical compatibility with biological tissues, and potential toxicity associated with heavy metal constituents.^8^ Organic polymers represented by polyvinylidene fluoride (PVDF) possess superior flexibility and lower toxicity. Nonetheless, their non-biodegradable property usually requires secondary surgical excision post-implantation.^9^ Furthermore, long-term retention of such materials *in vivo* can trigger chronic inflammation and foreign body response.^10^ These limitations have spurred interest in biodegradable and biocompatible piezoelectric materials derived from biomolecules.

Among biomolecular piezoelectric materials, amino acids and peptides stand out owing to their inherent chirality, self-assembly capability, chemical tailorability, and excellent biodegradability. Most amino acids are chiral and tend to crystallize in non-centrosymmetric space groups, giving rise to piezoelectricity.^7^^,^^11^ Glycine is the sole achiral proteinogenic amino acid; its β- and γ-polymorphs exhibit excellent piezoelectricity owing to their non-centrosymmetric space groups (P2_1_ and P3_2_, respectively).^12^^,^^13^ Moreover, non-covalent interactions such as hydrogen bonding and π-π stacking between peptide molecules enable the formation of ordered supramolecular structures with enhanced piezoelectric properties. Importantly, these biomolecular materials can degrade into naturally metabolizable small molecules, thereby circumventing the risks associated with long-term implantation and eliminating the need for secondary surgical intervention.^14^^,^^15^ For example, sensors fabricated from fluorinated N-Cbz-L-phenylalanine (Z-Phe) crystal films left only minimal debris after six weeks of subcutaneous implantation, with surrounding tissues displaying clear signs of healing.^16^

The biological significance of the piezoelectric effect in amino acids and peptides is intimately linked to naturally occurring piezoelectric architectures in biological tissues. Collagen fibers, the predominant structural protein in mammals, possess a non-centrosymmetric triple-helical structure that gives rise to electrical polarization upon mechanical deformation. As a result, collagen-rich tissues, including bone, cartilage, and ligaments, all exhibit piezoelectric properties.^3^^,^^17^^,^^18^ These bioelectrical signals have been shown to be involved in stem cell migration, wound healing, and tissue regeneration. For instance, the tibia generates a piezoelectric potential of approximately 300 μV during walking,^11^ which regulates bone remodeling and growth. At the cellular level, the electric field arising from piezoelectric performance guides stem cell migration toward injury sites via electrotaxis,^19^ thereby facilitating tissue repair. Inspired by the piezoelectricity inherent in native tissues, researchers have increasingly explored amino acid- and peptide-based materials for biomedical applications.

This review elucidates how amino acid chirality governs the directionality and specificity of supramolecular organization, and how the intrinsic dipole moments of chiral amino acids determine molecular-level polarization and final piezoelectric anisotropy of assembled products. We also provide a systematic comparison of different chemical modification approaches in terms of their efficiency in optimizing the piezoelectric performance of amino acids, including fluorination, functional group substitution, hydrogen-bonding induction effects, and metal coordination. Furthermore, we also compare the effects of different external-field-assisted processing methods, including electric field, thermal annealing, and mechanical force on the morphology, crystal structure, and piezoelectric response of FF assemblies. Additionally, we discuss biomedical applications, unresolved challenges, and future priorities, establishing a foundation for rational design of high-performance peptide-based piezoelectric materials.

## Advantages of natural piezoelectric materials

Conventional piezoelectric materials such as ceramics and polymers have been widely deployed in sensing, actuation, energy conversion, and catalysis. However, most piezoelectric implants are fabricated from permanent materials such as PZT or PVDF. Upon implantation, these non-biocompatible materials provoke excessive immune responses that prevent biointegration.^10^^,^^20^ Additionally, the high elastic modulus of piezoelectric ceramics creates a pronounced mechanical mismatch with soft biological tissues.^21^^,^^22^^,^^23^ Under load-bearing conditions, this leads to stress shielding-induced atrophy and resorption of adjacent bone tissue.^24^ Piezoelectric polymers offer advantages over ceramics in mechanical flexibility, reduced stiffness mismatch, and biocompatibility. However, their piezoelectric strain coefficients are typically modest; for instance, the d_33_ of PVDF is generally limited to 20–34 pC/N.^25^ Additionally, they exhibit insufficient mechanical durability under cyclic loading, being susceptible to interfacial delamination and surface cracking.^26^^,^^27^^,^^28^^,^^29^

By contrast, amino acid- and peptide-based piezoelectric materials are inherently flexible, biocompatible, and biodegradable. Upon hydrolytic or enzymatic degradation, they yield metabolites that are readily absorbed or excreted,^14^ effectively overcoming the biosafety and degradability limitations of traditional piezoelectrics. For example, a degradable piezoelectric nanogenerator fabricated by embedding diphenylalanine (FF) microrods within a polylactic acid (PLA) matrix undergoes complete dissolution in phosphate-buffered saline, acidic, and alkaline solutions after 25 days at 60 °C.^15^ Cheng et al. fabricated degradable isoleucine-based film devices capable of monitoring dynamic physiological activities, including muscle contraction and pulmonary respiration, with degradation exceeding 60% within six weeks.^6^ Amino acid- and peptide-based nanomaterials exhibit excellent biocompatibility. Taking FF-based nanocomposites as an example, fibroblasts seeded onto these materials achieve 80% viability within three days, indicating that the FF-based surface supports cell adhesion and proliferation without exhibiting significant cytotoxicity. This favorable biocompatibility renders them particularly suitable for implantable bioelectronic devices, ensuring enhanced *in vivo* safety.^30^ The Young’s modulus of self-assembled amino acid and polypeptide hydrogels can be tuned to the kilopascal range by adjusting self-assembly conditions, rendering it comparable to that of the extracellular matrix and thus achieving mechanical compatibility. For example, fluorene-methoxycarbonyl (Fmoc)-FF peptide hydrogels exhibit mechanical properties closely matching those of the extracellular matrix, facilitating tissue adhesion and signal transduction.^31^ Composite structures combining these materials with synthetic polymer shells such as polycaprolactone further augment mechanical and piezoelectric performance, enhancing tissue adhesion, cell proliferation, and angiogenesis while minimizing foreign body reactions.^32^ Piezoelectric amino acids and peptides exhibit unique advantages in self-assembly, manifested in the spontaneous nature of the assembly process, the optimization of supramolecular geometric packing structures, and the tunability of their resulting morphologies. Amino acids with non-centrosymmetric structures and peptides with specific sequences can spontaneously assemble into highly ordered supramolecular structures via non-covalent interactions, including hydrogen bonding, π-π stacking, and hydrophobic effects. Amino acids and peptides serve as molecularly programmable building blocks that enable direct tuning of molecular dipole moments. For instance, Cheng et al. replaced the hydrogen atoms on the aromatic rings of L-tryptophan, L-phenylalanine, and Z-Phe with fluorine. This fluorination significantly enhances polarization by increasing molecular dipole moments, optimizing supramolecular geometric packing structures, narrowing the bandgap, and improving charge mobility. Consequently, these fluoro-substituted crystals exhibit piezoelectric coefficients as high as 50.36 pm/V.^16^ Self-assembly further endows piezoelectric materials with a wealth of supramolecular architectures. For instance, FF is capable of self-assembling in solution into multiple morphologies, such as nanospheres, nanotubes, and nanofibers.^13^

Unlike conventional piezoelectric ceramics, which typically require high electric fields and elevated temperature for poling, peptides can be uniformly polarized at substantially lower electric field strengths, approximately 200 kV/m, which is significantly lower than that needed for ceramics.^33^ When intrinsic molecular dipoles are packed in a non-centrosymmetric manner, the dipole moments within the unit cell do not fully cancel out, giving rise to spontaneous polarization. For polycrystalline assemblies, external-field-assisted fabrication technologies, including electric-field-assisted deposition, mechanical stress, and annealing, can macroscopically reorient the grains to achieve uniform alignment of the molecular dipoles, thus maximizing the net polarization and substantially improving the piezoelectric performance.^34^ Finally, unlike high-temperature sintering required for ceramics or toxic solvents used in polymer processing, many amino acid/peptide assemblies can be prepared via solution-based or vapour-phase methods under mild conditions.^35^ This not only reduces the environmental footprint but also facilitates seamless integration with flexible and biodegradable substrates, a critical advantage for transient bioelectronics.

**Figure 1:**
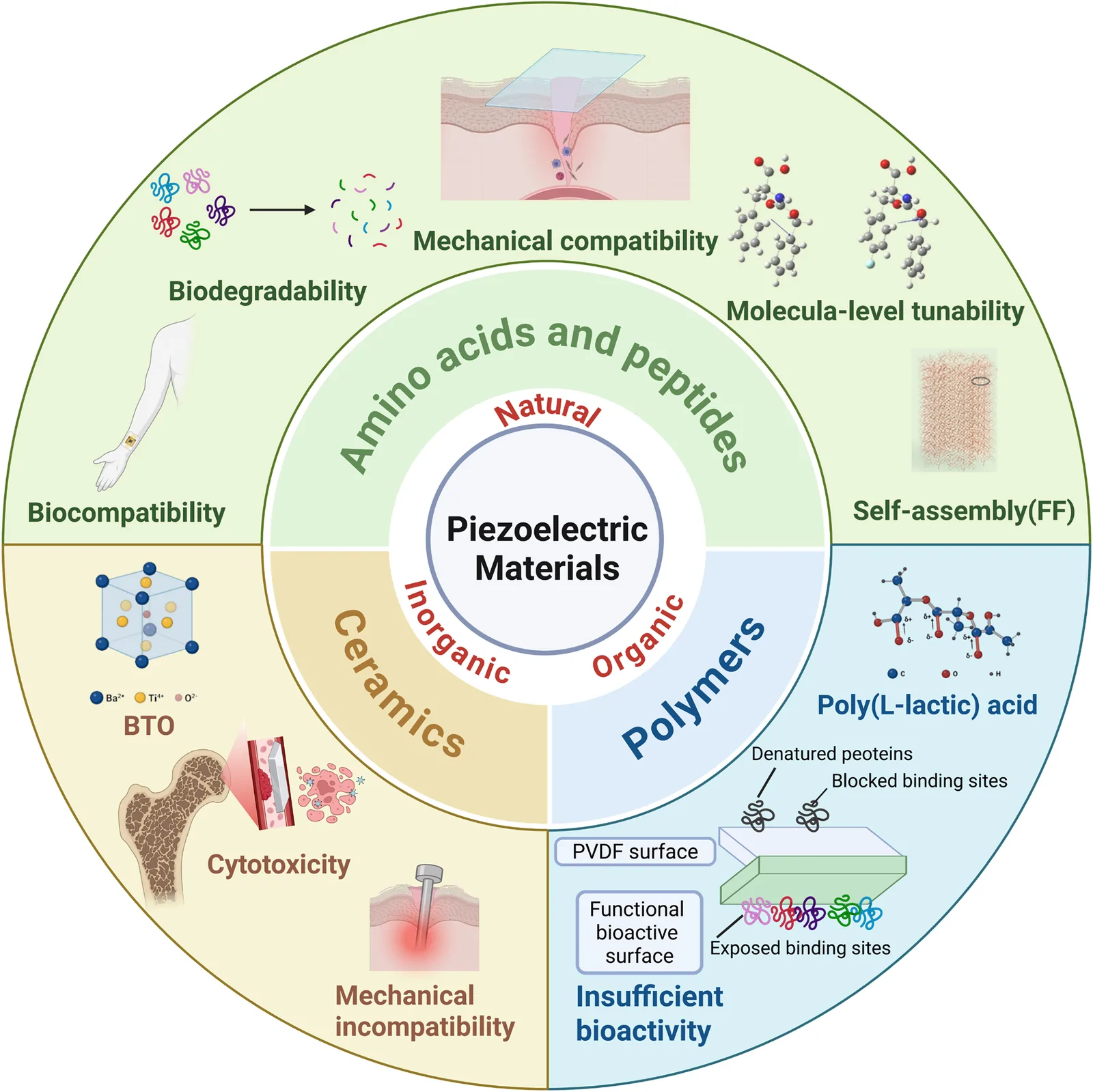
Comparison of biomedical application characteristics among piezoelectric amino acids/peptides, piezoelectric ceramics, and piezoelectric polymers^36^^,^^37^^,^^38^

Therefore, owing to their natural flexibility, biocompatibility, biodegradability, mechanical compatibility, self-assembly, low processing energy consumption, and environmental friendliness, amino acid- and peptide-based piezoelectric materials are suitable for applications in biosensing and tissue regeneration within living organisms, effectively overcoming the limitations of traditional materials (Figure 1).

## The mechanism of piezoelectricity in amino acids and peptides

The piezoelectric effect in amino acids and peptides originates from the molecular asymmetry and the inherent dipole moments associated with their polar functional groups. Upon mechanical deformation, the bond lengths and bond angles within the molecule undergo reversible changes, which in turn alter the magnitude or orientation of the dipole moments. Such variations drive the relative displacement of positive and negative charge centers. In crystals with non-centrosymmetric structures, the cooperative modulation of individual molecular dipole moments under mechanical stress can sum constructively to generate macroscopic polarization, giving rise to a measurable piezoelectric voltage at the material surface.^39^

**Figure 2:**
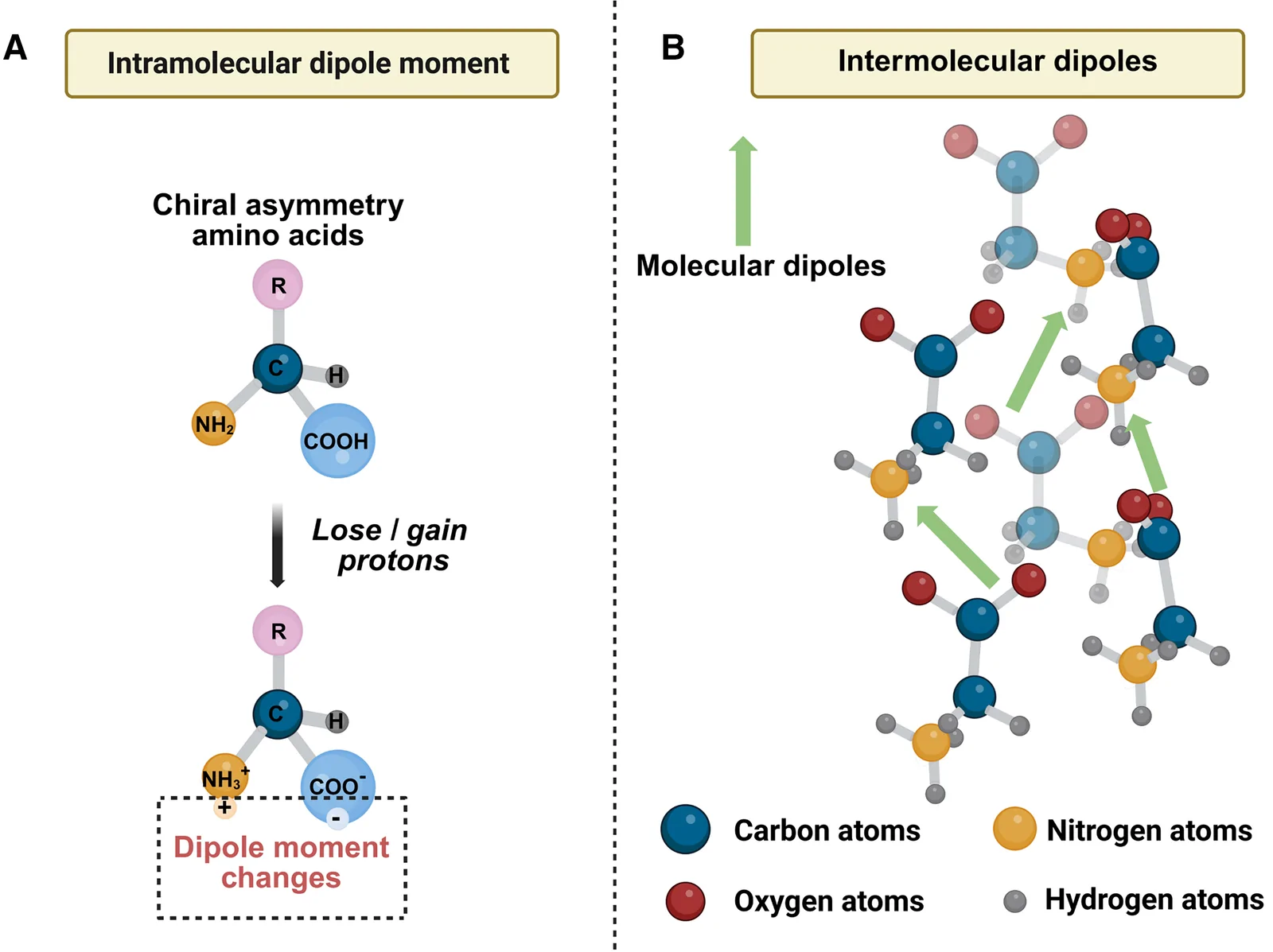
Intramolecular dipole moments and intermolecular dipole distributions in piezoelectric amino acids (A) Intramolecular dipole moment of amino acids. (B) Intermolecular dipoles in β-glycine crystallized in a monoclinic space group.

Amino acids, the fundamental structural units of proteins, consist of an amino group (-NH_2_), a carboxyl group (-COOH), and a characteristic side chain (R group).^40^ The piezoelectric effect in amino acids originates from the inherent molecular dipole moments generated by the electronegativity difference between the functional groups. Although the dipole moment of an individual amino acid molecule is too weak to produce a detectable piezoelectric response, the directional alignment of a large number of amino acid molecules within a non-centrosymmetric crystal lattice results in a summation of dipole moments, giving rise to a macroscopic piezoelectric output.^11^^,^^41^ Among the 20 proteinogenic amino acids, 19 are chiral and crystallize preferentially in non-centrosymmetric space groups,^12^ endowing them with piezoelectric properties,^7^^,^^42^ representative examples include L-threonine, L-hydroxyproline, and L-alanine. Although glycine is achiral, its β-polymorph also adopts a non-centrosymmetric crystal structure, conferring piezoelectric activity^7^ (Figure 2).

Peptides are chain polymers in which amino acid residues are linked by peptide bonds. As with amino acids, their piezoelectricity originates from internal molecular dipole moments^11^; however, the primary contribution is the polar amide group [─NH─C(═O)─], which gives rise to a net molecular dipole moment of 3.7 Debye due to the electronegativity difference between the O═C and N─H bonds in α-helical peptides.^43^ Since the dipole moment of a single peptide bond is small, macroscopic piezoelectric effects require specific spatial arrangements. In α-helices, hydrogen bonds are aligned in a highly ordered and unidirectional manner, generating a significant macroscopic dipole moment; in β-sheets, the hydrogen-bonded network organizes the peptide strands into extended sheets, where the collective alignment of backbone dipoles along the strand direction yields a net dipole, with its magnitude depending on whether the arrangement is parallel or antiparallel.^44^ External mechanical forces that stretch, bend, or reorganize these hydrogen bond networks alter the orientation of the dipole moments, producing net polarization and enabling mechanical-to-electrical energy conversion.^45^ Moreover, peptides can be driven to self-assemble into ordered supramolecular architectures including nanotubes and nanofibers through cooperative noncovalent interactions such as hydrogen bonding, van der Waals forces, electrostatic interactions, hydrophobic interactions, and π-π stacking.^4^^,^^46^ This self-assembly process produces high molecular alignment along the long axis in a non-centrosymmetric arrangement, with structural stability and polarization synergy further reinforced by hydrogen bond networks and π-π stacking, yielding an enhanced macroscopic piezoelectric response.^47^

**Figure 3:**
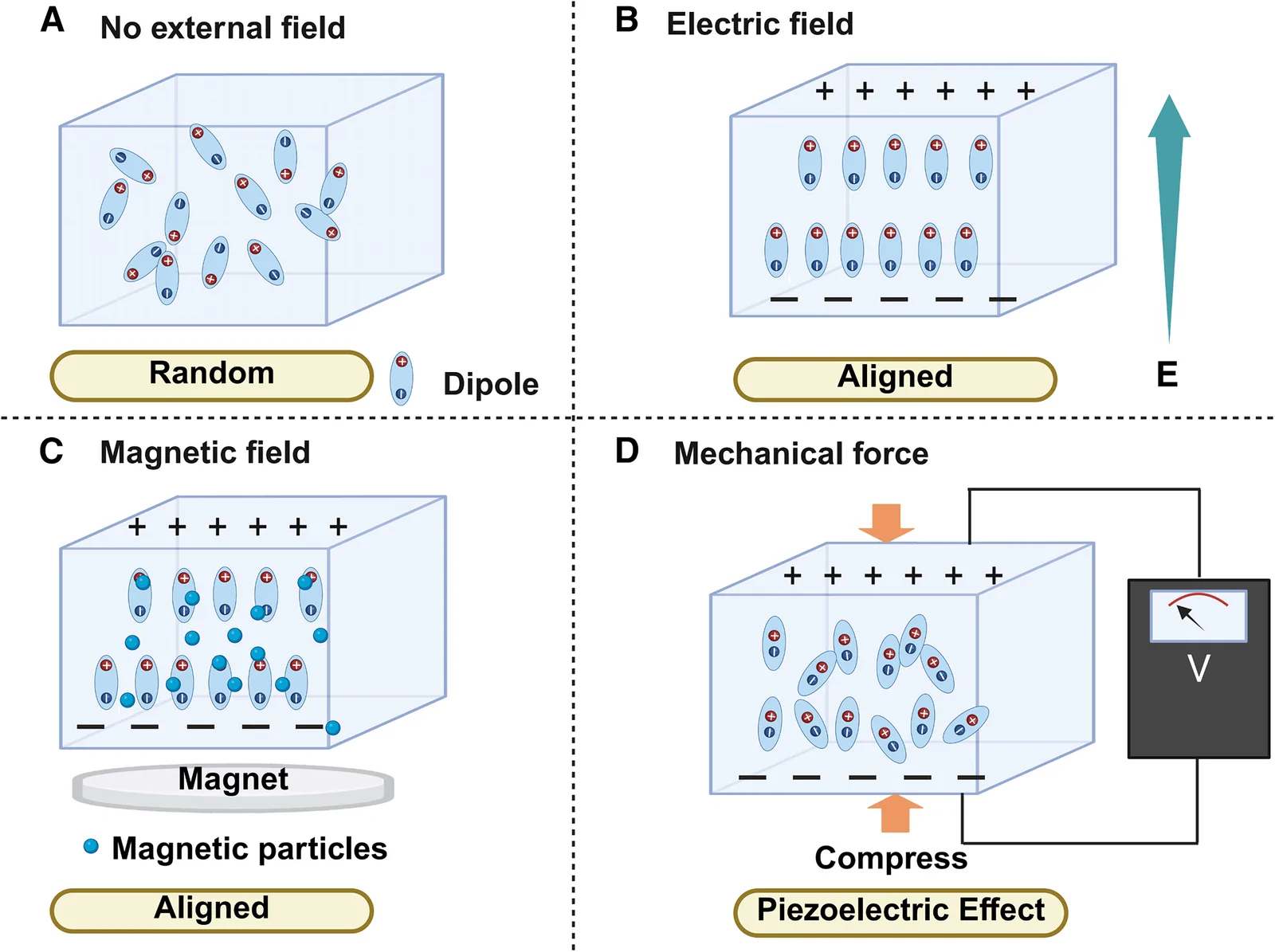
Distribution of intermolecular dipole moments of amino acids and peptides under natural conditions and under electric, magnetic, and mechanical fields (A) Dipole distribution in space under ambient conditions. (B) Dipoles align in the same direction under an applied electric field. (C) Dipoles align unidirectionally under a magnetic field upon co-assembly of amino acids with magnetic particles. (D) Mechanical stress induces a relative displacement between positive and negative charge centers within the crystal or molecule. Dipole moments aligned in the same direction constructively superimpose, giving rise to macroscopic polarization, which generates a measurable piezoelectric voltage on the material surface.

For peptide materials, their piezoelectric properties depend not only on molecular conformation but also on higher-order structural factors. Even with the same secondary structure, such as α-helices or β-sheets, different peptides may still exhibit significant differences in their piezoelectric coefficients, which could arise from the combined effects of intramolecular hydrogen bond geometry, intermolecular packing arrangements, and side-chain chemical environments. Firstly, geometric parameters of the hydrogen bond network, such as the bond angle and bond length of [─NH─C(─O)─],^48^^,^^49^ directly influence the orientation and magnitude of the amide dipole. The precise geometric parameters of peptide helices/sheets, such as the rise angle, pitch, and the number of residues per turn in an α-helix, determine the projection components of the molecular dipole along specific crystal axis directions.^50^ Secondly, intermolecular packing patterns, such as parallel and antiparallel arrangements of β-sheets, determine whether adjacent molecular dipoles coherently add up or cancel each other out, thus regulating the macroscopic polarization strength.^51^ Additionally, side-chain chemical modifications, such as hydroxylation, fluorination, or the introduction of N-terminal protecting groups (such as Fmoc), can further modulate the piezoelectric response by altering the intermolecular π-π stacking strength, hydrophobic interactions, and the distribution of electrostatic potential. These multiscale structural factors collectively determine the anisotropic characteristics of the piezoelectric tensor in peptides, providing a theoretical basis for the rational design of piezoelectric properties (Figure 3).

## Classification of piezoelectric amino acids and peptides

### Piezoelectric amino acids

Based on crystal symmetry, piezoelectric amino acids can be broadly divided into chiral and achiral categories. The 19 chiral proteinogenic amino acids excluding glycine typically crystallize in non-centrosymmetric space groups, wherein the absolute configuration at the chiral α-carbon constrains the torsional degrees of freedom of the side chain and the ammonium/carboxylate head groups, thereby dictating the hydrogen-bonding network topology and the relative orientation of molecular dipoles within the unit cell, and conferring piezoelectric activity.^7^^,^^12^^,^^13^ The piezoelectric activity of amino acids is governed by non-centrosymmetric crystal packing and can be further modulated through hydrogen bonding networks.

Chiral configuration is a key factor governing the molecular stacking patterns and macroscopic piezoelectric performance of amino acid crystals. The 19 proteinogenic amino acids are chiral and preferentially crystallize in Sohncke space groups, which lack inversion symmetry and are thus compatible with piezoelectricity. Single-enantiomer L-amino acids typically crystallize in such non-centrosymmetric space groups.^14^^,^^52^ For example, in single-crystal form, L-threonine, L-hydroxyproline, and L-alanine crystallize in the non-centrosymmetric space group P2_1_2_1_2_1_. The three mutually perpendicular 2-fold axes of this nonpolar point group permit shear piezoelectric coefficients (d_14_, d_25_, d_36_) arising from non-centrosymmetric molecular arrangements, yet forbid the longitudinal coefficient d_33_ due to the absence of a unique polar axis. Consequently, these amino acids exhibit piezoelectric activity exclusively under shear deformation, with no measurable longitudinal response to compressive or tensile stress along the principal crystallographic axes.^53^ By contrast, L-isoleucine crystallizes in the polar space group P2_1_. The unique polar axis along the 2-fold screw axis nominally permits the longitudinal piezoelectric coefficient d_33_. The molecular dipoles of L-isoleucine are predominantly distributed within the bc-plane, constrained by an extensive hydrogen-bonding network involving seven hydrogen bonds per molecule. This in-plane dipole orientation limits the c-axis projection of the molecular dipole, thereby suppressing the longitudinal response. Instead, shear deformation within the bc-plane induces significant dipole reorientation, yielding an optimal shear piezoelectric coefficient d_34_ of 25 pC/N. Consequently, despite the polar nature of the P2_1_ space group, L-isoleucine exhibits its strongest piezoelectric activity under shear deformation, with the longitudinal response remaining suboptimal.^12^ The strength of the hydrogen-bond network is a critical determinant of piezoelectric performance in chiral crystals. For L-methionine, relatively weak hydrogen bonding results in a loosely packed molecular arrangement. Under mechanical stress, this arrangement facilitates a large electric dipole moment, manifesting as a high piezoelectric coefficient (*d*_22_ = 37.6 pm/V).^54^

DL-Alanine, a representative racemic amino acid piezoelectric crystal, crystallizes in the polar space group Pna2_1_, where glide planes perpendicular to the a- and b-axes, combined with the 2-fold screw axis along the c-direction, establish a unique polar axis that permits the longitudinal piezoelectric coefficient d_33_. Within this structure, D- and L-alanine molecules alternate in the ab-plane yet adopt parallel dipole alignments along the polar c-axis in each successive layer, thereby preserving a net macroscopic polarization rather than canceling it. This architecture is common to all crystal structures in polar space groups such as Pna2_1_, where molecular dipoles are parallel or nearly parallel to the polar axis; as a consequence, the unit cell possesses a nonzero dipole moment.^55^ This cooperative heterochiral packing yields a theoretical longitudinal coefficient d_33_ of 10.3 pC/N and an experimentally measured value of approximately 4.8 pC/N, demonstrating superior longitudinal piezoelectric performance compared to its single-enantiomer counterparts.^56^^,^^57^

The piezoelectricity of achiral glycine depends on its polymorph. Among glycine polymorphs, the centrosymmetric α-glycine exhibits no piezoelectricity, while doping of centrosymmetric α-glycine crystals with other L-amino acids also leads to crystals with piezoelectric properties.^58^ While β-glycine (space group P2_1_) exhibits an exceptionally high shear piezoelectric coefficient (*d*_16_ = 178 pm/V), it is metastable. In contrast, γ-glycine (space group P3_2_) generates piezoelectricity through net polarization in the a-b plane with a longitudinal piezoelectric coefficient *d*_33_ = 9.93 pm/V and is relatively stable.^12^^,^^13^ In this non-centrosymmetric structure, lattice compression enhances polarization, whereas stretching weakens hydrogen bonding forces and reduces polarization.^59^^,^^60^

In summary, the piezoelectricity of amino acids fundamentally originates from non-centrosymmetric crystal structures. Chiral amino acids are piezoelectric by virtue of their non-centrosymmetric space groups, while the β- and γ-polymorphs of achiral glycine display excellent piezoelectricity owing to their polar space groups. Racemic compounds like DL-alanine also exhibit piezoelectric responses through their non-centrosymmetric crystal packing. These intrinsic piezoelectric properties endow amino acids with significant potential as building blocks for bio-piezoelectric devices.

### Piezoelectric peptides

Peptides are short chains composed of amino acid monomers linked by peptide bonds. Based on molecular configuration and secondary structure, piezoelectric peptides can be classified into three main categories: α-helical peptides, β-sheet peptides, and cyclic peptides.

In α-helical peptides, hydrogen bonds oriented parallel to the helix axis generate aligned electric dipole moments; the cooperative alignment of these dipoles along the helical axis gives rise to a piezoelectric response. Poly(γ-benzyl L-glutamate) (PBLG) is a prototypical α-helical polypeptide whose intramolecular hydrogen bonds are directionally arranged along the helical axis, producing a macroscopic dipole moment. Its non-centrosymmetric structure enables piezoelectric output under an applied electric field or shear stress.^61^ By controlling the electric field intensity and fiber orientation during electrospinning, PBLG fibers with a high piezoelectric coefficient (*d*_33_ = 25 pC/N) and favorable thermal stability have been obtained, rendering them suitable for applications including controlled drug delivery.^62^^,^^63^

The piezoelectricity of β-sheet polypeptides originates primarily from the non-centrosymmetric arrangement of peptide bond dipoles within the β-sheet layers. The directionality of interchain hydrogen bonds and the intrinsic twist of the sheets disrupt inversion symmetry, generating net polarization under mechanical stress.^64^ Upon self-assembly into nanofibers, the cumulative twist of the β-sheets, combined with electrostatic contributions from side chains, produces polarization summation along the fiber axis.^65^ FF is a canonical example of β-sheet peptides. Composed of two phenylalanine units, it is the shortest known self-assembling peptide sequence. Through intermolecular interactions, FF readily self-assembles into well-ordered nanostructures, notably peptide nanotubes.^4^^,^^66^ Molecular simulations indicate that the initial FF aggregation in solution is driven by electrostatic forces, followed by solvent-mediated growth into ordered nanotubes.^67^^,^^68^ These nanotubes adopt a non-centrosymmetric hexagonal crystal structure (space group P6_1_), intermolecular π-π stacking, and hydrogen bonding interactions along the tube axis, generating high shear piezoelectricity (d_15_). FF also exhibits high biocompatibility, pyroelectricity, and ferroelectricity, making it one of the most extensively studied piezoelectric peptides.^13^^,^^40^ Of its four independent piezoelectric coefficients (d_33_, d_31_, d_15_, d_14_), d_33_ typically dominates the piezoelectric response,^69^ reaching 9.9 pC/N in film form^70^^,^^71^

The piezoelectric properties of cyclic peptides are governed by unidirectional hydrogen bonding-driven stacking of the cyclic ring motifs.^72^ Cyclic dipeptides can self-assemble through noncovalent interactions.^72^^,^^73^ In particular, tryptophan-containing cyclic dipeptides such as cyclo-phenylalanine-tryptophan (cyclo-FW) and cyclo-glycine-tryptophan (cyclo-GW) form supramolecular assemblies through hydrogen bonding and interactions, significantly enhancing piezoelectric performance^74^ (Table 1; Table 2).

**Table 1:** The piezoelectric principles and commonly used coefficients of typical piezoelectric amino acids and peptides

Type		Piezoelectric principle	Materials		Piezoelectric coefficient	Reference
Amino acid	chiral amino acids	chiral asymmetric molecular structure	L-methionine	single crystals	*d*_22_ = 37.6 pm/V	Yuan et al.^75^
			L-isoleucine	single crystals	*d*_34_ = 25 pm/V	Guerin et al.^76^
			L-valine	single crystals	*d*_22_ = 12.3 pm/V,*d*_23_ = 0.05 pC/N,*d*_34_ = 1.81 pC/N	Yuan et al.^75^
			L-arginine	single crystals	*d*_36_ = 3.64 pC/N,*d*_14_ = 0.28 pC/N	Guerin^53^
			L-glutamic	single crystals	*d*_36_ = −3.18 pm/V,*d*_14_ = 0.54 pC/N,*d*_25_ = −1.60 pC/N	Guerin et al.^7^
			L-threonine	single crystals	*d*_36_ = −4.90 pC/N,*d*_14_ = 3.78 pC/N,*d*_25_ = −3.40 pm/V	Guerin et al.^7^
			L-tyrosine	single crystals	*d*_25_ = −9.73 pC/N,*d*_14_ = −5.00 pC/N,*d*_36_ = −5.81 pC/N	Guerin et al.^7^
			L-glutamine	single crystals	*d*_36_ = −11.40 pC/N,*d*_14_ = −1.85 pC/N,*d*_25_ = −3.78 pC/N	Guerin et al.^7^
			L-leucine	single crystals	*d*_36_ = 42.2 pm/V,*d*_34_ = 20 pm/V	Yuan et al.^75^; Guerin et al.^76^
			L-hydroxyproline	single crystals	*d*_25_ = −27.75 pC/N,*d*_14_ = 3.72 pC/N,*d*_36_ = 4.55 pC/N	Guerin et al.^7^
				nanocrystalline films	*d*_33_ = ±1 pC/N	Guerin et al.^7^
			L-alanine	single crystals	*d*_36_ = −6.30 pC/N,*d*_14_ = −6.26 pC/N,*d*_25_ = −3.78 pC/N	Guerin et al.^7^
	Achiral amino acids	non-centrosymmetric crystal form	β-glycine	single crystals	*d*_16_ = 178 ± 11 pm/V,*d*_22_ = −5.7 pm/V	Guerin et al.^76^
				nanocrystalline films	*d*_33_ = 11.2 pm/V	Zhang et al.^77^
			γ-glycine	single crystals	*d*_33_ = 9.93 pm/V,*d*_16_ = 6 pm/V,*d*_11_ = 1.7 pm/V,*d*_22_ = −1.1 pm/V	Guerin et al.^76^
	External racemate complex	non-centrosymmetric crystal form	DL-alanine	single crystals	*d*_33_ = 10.3 pC/N	Ghosh et al.^78^
				nanocrystalline films	*d*_33_ = 4.8 pC/N	Guerin et al.^79^
Peptide	α-helical structure polypeptide	hydrogen bonds arranged parallel to the helix axis generate a high electric dipole moment	PBLG	nanofibers	*d*_33_ = 25 pC/N	Farrar et al.^80^
				films	*d*_15_ = 18 pC/N,*d*_33_ = 23 pC/N	Uehara et al.^81^; Farrar et al.^82^
			Poly(γ-methyl L-glutamate) (PMLG)	films	*d*_14_ = 2 pm/V,*d*_33_ = 23 pm/V	Farrar et al.^82^
	β-sheet structured polypeptide	π-π stacking and hydrogen bonding interactions	FF	nanotubes	*d*_15_ = 80 pm/V,*d*_33_ = 18 pC/N,*d*_14_ = −10 pm/V,*d*_31_ = 4 ± 1 pm/V	Vasilev et al.^69^
				vertically aligned microrod array	*d*_33_ = 17.9 pm/V	Nguyen et al.^83^
			Fmoc-FF	nanotubes	*d*_15_ = 33.7 ± 0.7 pm/V	Ryan et al.^84^
				nanofibers	*d*_15_ = 1.7 ± 0.5 pm/V	Ryan et al.^84^
	Circular peptide	π-π stacking and hydrogen bonding interactions; the closed-loop structure enhances stability, mechanical properties, and piezoelectricity	cyclo-GW	crystal powder	*d*_36_ = 14.1 pC/N	Tao et al.^85^
			cyclo-FW	crystal powder	*d*_eff_ = 16 pm/V	Tao et al.^74^; Santos et al.^72^

**Table 2:** Structural and piezoelectric parameters of amino acids and peptides

Category	Representative molecule	Molecular configuration	Space group	Dipole alignment	Piezoelectric coefficient	Reference
Achiral amino acids	α-glycine	achiral	P2_1_/c	centrally symmetric, with dipole moments canceling each other out	No macroscopic piezoelectricity	Guerin et al.^12^
	β-glycine	achiral	P2_1_	they crystallize into metastable polar phases, with the dipoles aligned in the same direction	*d*_16_ = 178 pm/V	Guerin et al.^12^
	γ-glycine		P3_2_		*d*_33_ = 10.4 pC/N	Liu et al.^20^
Racemic amino acid	DL-alanine	racemate	P2_1_	DL-alanine crystallizes with its molecular dipoles well aligned in both longitudinal and transverse directions	*d*_33_ = 10.3 pC/N	Guerin et al.^57^
Chiral amino acids	L-isoleucine	homochiral (L-form)	P2_1_	the dipole distribution is predominantly in the b-c plane	*d*_33_ = 1.2 pC/N	Cheng et al.^6^
α-helical structure polypeptide	PBLG	axial chirality	P6_1_	the C═O and N–H groups of the peptide bonds are aligned in the same direction along the helical axis, and the hydrogen-bonding network forms a strong axial cumulative dipole	*d*_33_ = 23 pC/N	Farrar et al.^82^
β-sheet structured polypeptide	FF (vertically aligned microrod array)	interchain chiral arrangement	P6_1_	the dipole moments are oriented along the electric field, and the adjacent peptide chains are arranged in the same direction	*d*_33_ = 17.9 pm/V	Nguyen et al.^86^
Circular peptide	cyclo-GW	cyclic chiral configuration	P2_1_	the dipole distribution is predominantly in the a-b plane	*d*_36_ = 14.1 pC/N	Tao et al.^87^

Owing to their distinctive molecular structures, hydrogen-bonding arrangements, and self-assembly behaviors, α-helical, β-sheet, and cyclic peptides generate piezoelectric effects. Representative peptides (e.g., FF) combine superior piezoelectric properties with favorable biocompatibility, making them promising for biomedical applications.

## Enhancing the piezoelectricity of amino acids and peptides through molecular modification

Although amino acids inherently possess piezoelectric properties, their piezoelectricity is weaker than that of inorganic and polymeric piezoelectric materials, and they are prone to degradation. To enhance the piezoelectric performance and physicochemical properties, various molecular modification strategies have been developed, including chemical functionalization, microstructural regulation, hydrogen-bonded heterostructure engineering, and dopant incorporation.^46^^,^^88^

### Chemical modification

Due to the outstanding electronegativity of fluorine and the high polarity of the C-F bond, as well as the presence of numerous piezoelectric fluorinated polymers, introducing F into piezoelectric biomaterials represents a straightforward and effective strategy to enhance piezoelectricity. Hu et al. improved the piezoelectricity of phenylalanine derivatives by fluorinating their side chains. Among the resulting materials, Cbz-Phe (4F) crystallized in the C_2_ space group and exhibited a piezoelectric coefficient d_33_ of 17.9 pm/V.^89^ Xue et al. performed aromatic ring fluorination on Z-Phe, significantly amplifying the piezoelectric effect by increasing the molecular dipole moment, optimizing supramolecular stacking, and reducing the bandgap. The resulting fluorinated Z-Phe crystals achieved a piezoelectric coefficient of 50.36 pm/V, substantially exceeding the 23.59 pm/V of the unfluorinated analogue.^37^

Functional group modification represents another viable approach. Acetylation of amino acids increases the polarization of their supramolecular arrangement, thereby enhancing piezoelectricity. Natural L-tryptophan exhibits no observable piezoelectric effect, whereas acetylated tryptophan (L-AcW) displays a predicted maximum piezoelectric strain tensor of up to 47 pm/V.^90^ Introducing additional aromatic moieties such as phenyl, porphyrin, naphthalene, and Fmoc groups can further reinforce π-π stacking interactions. Gazit et al. designed tert-butyloxycarbonyl (Boc)-beta-diphenyl-Ala-OH (Dip)-Dip based on FF, doubling the number of aromatic residues to form a denser aromatic network with an effective piezoelectric coefficient d_33_ reaching 73.1 ± 13.1 pC/N.^91^ Fmoc modification of FF promotes self-assembly into a non-centrosymmetric structure, affording d_15_ values of 33.7 pm/V and 1.7 ± 0.5 pm/V for nanotubes and nanofibers respectively, making these assemblies attractive for tissue engineering scaffold applications.^84^^,^^92^ Even simple modifications such as hydroxylation can modulate piezoelectric output. Thompson et al. demonstrated that under equivalent strain, the polarization of L-hydroxyproline is significantly greater than that of L-proline, with the piezoelectric constant rising from <1 pC/N to 28 pC/N.^93^

Overall, modifications at the molecular level can effectively enhance piezoelectricity by tuning the charge distribution, dipole alignment, and supramolecular packing.

### Hydrogen bonding induction effects at heterogeneous interfaces

Modulation of intermolecular hydrogen bonding can substantially alter a material’s microstructure and induce crystal reorganization, leading to enhanced piezoelectric coefficients.^94^ A widely adopted approach involves forming hydrogen bond networks between the polymer matrix and functional groups of amino acid molecules, coupling flexible polymers with piezoelectric amino acid crystals into a “hydrogen-bonded heterostructure”. In this system, hydrogen bonds act as physical crosslinking points to enhance mechanical toughness. They also serve as structural templates that direct the alignment of amino acid molecules along specific directions, thereby promoting the formation of non-centrosymmetric piezoelectric crystal phases. For example, DL-alanine complexed with PVA forms a hydrogen bond upon complexation and, at a component ratio of 1:3, exhibits piezoelectric performance comparable to that of γ-glycine.^95^ Yang et al. demonstrated that combining PVA with amino acids generates a dense hydrogen-bond network between the hydroxyl groups of PVA and the amino and carboxyl groups of glycine. This endows the PVA-glycine composite film with both mechanical flexibility and piezoelectricity while retaining good biodegradability, making it suited for biomechanical motion sensing.^96^ In general, the hydrogen-bonding network between the polymer matrix and amino acid molecules can guide crystal orientation, enhance dipole alignment, and improve piezoelectric output, providing a versatile means of simultaneously optimizing flexibility, piezoelectric performance, and biodegradability.

### Modifying single-molecule systems

Single-molecule systems typically exhibit limited piezoelectric performance owing to their relatively uniform charge distribution. However, piezoelectric performance can be enhanced by modulating intramolecular structures or introducing exogenous components to increase structural asymmetry.

**Figure 4:**
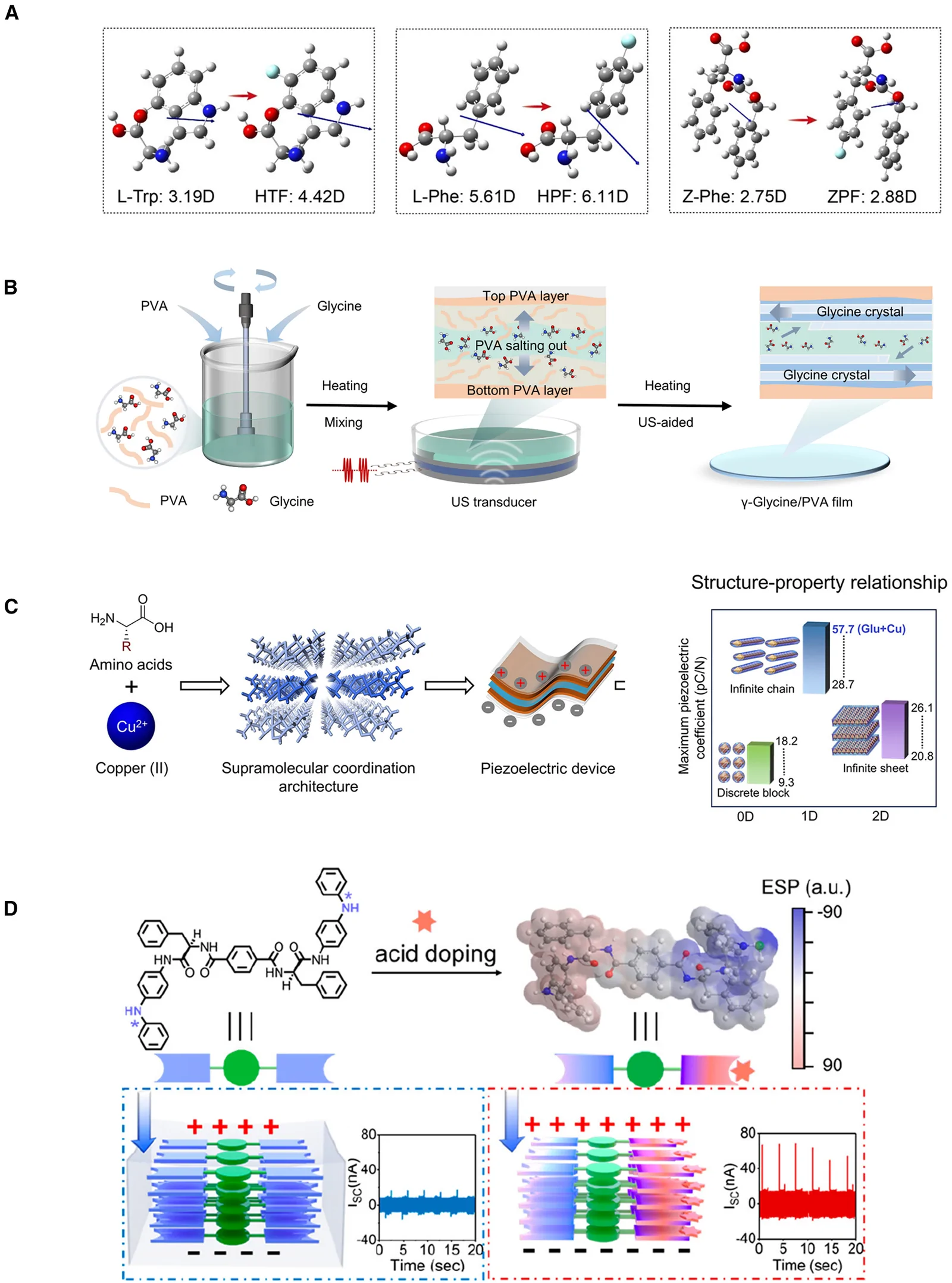
Molecular engineering of piezoelectric biomaterials (A) Calculated molecular dipole moments of L-tryptophan and fluoro-substituted derivatives of L-tryptophan (HTF), L-phenylalanine and fluoro-substituted derivatives of L-phenylalanine (HPF), and Z-Phe and fluoro-substituted derivatives of Z-Phe (ZPF).^16^ (B) Flexible, biodegradable ultrasonic wireless electrotherapy device based on highly self-aligned piezoelectric biofilms; hydrogen bonds are formed between different materials.^59^ (C) Certain amino acids could form complexes with copper (II), where the chiral amino acids used were the L-form. Copper (II) amino acid complexes have diverse coordination environments and self-assemble into tunable dimensional coordination networks (0D-2D), forming crystalline structures through supramolecular interactions. The differences in the dimensions of coordination networks and supramolecular stacking patterns modulate the piezoelectricity of copper (II) amino acid crystals^100^ (Adapted from Liu et al.^100^ with permission). (D) Acid doping can increase the asymmetric distribution of charges in the molecules and in turn molecular polarizability, leading to enhanced molecular piezoelectricity of assemblies^101^ (Adapted from Xia et al.^101^ with permission).

In terms of intramolecular structural engineering, metal coordination or chemical modification can directly alter the electron distribution and spatial configuration, thereby optimizing polarization. Coordination of Cu^2+^ with L-glutamic acid induces the formation of a helical structure, achieving unidirectional dipole moment alignment and increasing the piezoelectric coefficient to 57.7 pC/N.^97^ Acid doping of D-phenylalanine derivatives can similarly augment charge asymmetry, raising the effective piezoelectric coefficient to 38.5 pm/V, approximately four times that of the undoped material.^98^ At the intermolecular level, the introduction of exogenous molecules can break the symmetry of the original centrally symmetric crystal and induce piezoelectric effects. For example, α-glycine is intrinsically centrosymmetric and non-piezoelectric; however, doping with L-threonine or L-alanine disrupts crystal symmetry, generating vertical and shear piezoelectric effects, respectively^99^(Figure 4; Table 3).

**Table 3:** Quantitative comparison of molecular modification approaches

Chemical modification method	Primordial molecule	Primordial molecular piezoelectric coefficient	Chemically modified molecule	Maximum piezoelectric coefficient	Mechanism	Reference
Fluorination	L-tryptophan	nearly undetectable	HTF	7.11 pm/V	the dipole moment of the molecule after fluoro-substitution is higher than that of the corresponding molecule without fluoro-substitution. ZPF molecules exhibit parallel stacking without significant rotation, leading to an additive alignment of molecular dipole moments and an increase in net polarization.	Cheng et al.^16^
	L-phenylalanine	nearly undetectable	HPF	30.79 pm/V		
	Z-Phe	23.59 pm/V	ZPF	50.36 pm/V		
Functional group substitution	L-tryptophan (acetylated amino acids)	nearly undetectable	L-AcW	47 pm/V	acetylation increased the consistency of the orientation of acetylated amino acid crystals due to the reduction of molecular dipole moments.	Wang et al.^90^
	FF nanotubes (increase the number of aromatic groups)	*d*_33_ = 18 pC/N	Boc-Dip-Dip	*d*_33_ = 73 pC/N	the doubled number of aromatic groups per unit, compared to FF, produced a dense aromatic zipper network.	Basavalingappa et al.^46^
Hydrogen Bonding Induction Effects	DL-alanine	*d*_33_ = 4.0 pC/N	PVA/DL-alanine	*d*_33_ = 5.3 pC/N	hydrogen bonds between the materials induce the amino acid molecules to arrange in a specific direction.	Guerin et al.^57^; Jeon et al.^102^
Metal coordination	L-glutamate	*d*_14_ = 0.54 pC/N	copper(II) l-glutamate	*d*_14_ = 57.7 pC/N	metal coordination alters the electronic distribution and spatial configuration of the molecule, causing the dipole moment to be arranged in a single direction and increasing the piezoelectric coefficient.	Guerin et al.^7^; Liu et al.^100^

## Methods for the synthesis of piezoelectric amino acids and peptides

### Electric field-driven fabrication techniques

Electric fields are widely employed in the large-scale organization of piezoelectric biomaterials owing to their ability to exert homogeneous forces on charged and intrinsically polar molecules. Beyond directing microfabrication and self-assembly, electric field poling serves as a pivotal strategy for achieving macroscopic polarization. By applying an *in situ* electric field during molecular assembly or film formation, random thermally driven dipole fluctuations are suppressed, and dipoles are collectively aligned along the field direction. This mechanism effectively translates microscopic piezoelectricity into a pronounced macroscopic piezoelectric response.^103^ Taking FF as an example, an applied electric field can induce a transition from a disordered to an extended conformation, with the adjacent arrangement of benzene rings and π-π stacking interactions further enhancing structural stability. Electric field application to FF nanotubes also induces lattice deformation, enabling regulation of nanotube expansion and contraction.^15^^,^^104^ Nguyen et al. applied an electric field during FF self-assembly to achieve uniform polarization of the peptide nanotubes, yielding a piezoelectric coefficient d_33_ of 17.9 pm/V, which far exceeds the previously reported value of 9.9 pm/V.^83^ Common electric field-based fabrication approaches include electrohydrodynamic spray deposition, corona discharge, and electrospinning.^82^^,^^105^

Electrohydrodynamic spray deposition exploits the synergistic effect of an *in situ* electric field and nanoconfinement to guide oriented particle alignment and promote uniform nucleation. β-Glycine films prepared by this technique achieved large-area fabrication of the metastable β-polymorph for the first time, with d_33_ reaching 11.2 pm/V, significantly outperforming most known bio-organic piezoelectric films.^77^ The corona discharge method uses a high-voltage electric field to align dipoles within a liquid phase, enabling polarization of the material and enhancement of macroscopic piezoelectric properties. For example, PBLG dissolved in methyl methacrylate monomer can be simultaneously polarized and cured by corona discharge, yielding a composite film with *d*_33_ = 23 pC/N.^82^^,^^106^ In electrospinning, a high-voltage electric field is applied between the spinneret and the collector, which causes the polymer droplet to form a Taylor cone at the needle tip and generate a high-speed jet at the cone tip. Under the combined action of axial stretching by the electric field force and mechanical force, the dipole moments are induced to align unidirectionally along the field direction to produce piezoelectric fibers.^91^^,^^107^^,^^108^ Electrospun PBLG fibers prepared under controlled field strength and fiber orientation exhibit a high piezoelectric coefficient *d*_33_ = 25 pC/N with good thermal stability.^63^^,^^80^ In summary, given that most piezoelectric amino acids and peptides are non-ferroelectric and therefore not amenable to post-fabrication poling, applying an electric field during fabrication is particularly important for inducing polarization alignment and maximizing piezoelectric output.

### Magnetic field-driven fabrication techniques

Analogous to the electric field, an applied magnetic field can induce directional alignment and polarization of molecular dipoles, generating macroscopic piezoelectricity.^103^^,^^109^ Many molecular materials are diamagnetic, and the alteration of electron orbital motion by an applied magnetic field produces a diamagnetic anisotropy that enables alignment under sufficiently strong fields.^109^^,^^110^ For instance, FF nanotubes undergo macroscopic ordering under strong magnetic fields (e.g., 12 T), whereas weak fields (<0.6 T) exert negligible influence on their self-assembly.^34^^,^^109^ Magnetic responsiveness can be substantially enhanced by incorporating ferro- or paramagnetic nanoparticles into these biomolecular assemblies. Feng et al. co-assembled a diphenylalanine derivative with surface-functionalized Fe_3_O_4_ magnetic nanoparticles, achieving highly ordered alignment under a magnetic field as low as 0.5 T and increasing the piezoelectric coefficient markedly from 10.4 pm/V to 121.9 pm/V.^111^ The aromatic rings in α-helical peptides also generate significant diamagnetic anisotropy that enables torque under magnetic fields. X-ray diffraction confirmed chain alignment perpendicular to the field direction, and PBLG films prepared under a 10 T magnetic field exhibited a high shear piezoelectric coefficient (d_14_ up to 26 pC/N), significantly surpassing values reported for unoriented or drawn PBLG films (<1 pC/N).^63^ Nonetheless, the application of magnetic fields in piezoelectric material fabrication remains less developed than electric field approaches and warrants further investigation.

### Mechanical force-driven fabrication techniques

Mechanical force provides a powerful platform for the microfabrication and assembly of piezoelectric biomaterials. The application of mechanical forces to non-solid-phase bio-crystals induces oriented polarization, thereby enhancing their piezoelectric properties. The core mechanism lies in exploiting the constraints generated by external forces or spatial confinement. These constraints drive the oriented alignment of biomolecules or crystals along specific directions, disrupting random orientation and achieving macroscopic accumulation of microscopic piezoelectric responses. Principal methods include meniscus force-induced self-assembly, mechanical induction under nanoconfinement, and mechanical annealing with stress-induced recrystallization.^6^^,^^112^^,^^113^

A meniscus refers to the curved liquid surface formed at the solid-liquid-air triple-phase contact line due to the combined effects of liquid surface tension, solid surface energy, and gravity. Capillary flow during substrate withdrawal induces an ordered arrangement of solute molecules. FF nanotubes with unidirectional alignment prepared by this technique achieved a high piezoelectric coefficient of 46.6 pm/V.^113^^,^^114^ Nanoconfinement-induced self-assembly restricts molecules within a nanoscale space, facilitating directed self-assembly under geometrically constrained conditions. This method was used to prepare elastic phenylalanine dipeptide crystal fibers (FF-CFs), enabling FF crystals to rapidly self-assemble within a styrene-block-butadiene-block-styrene (SBS) matrix into a unique Mortise-Tenon structure with a longitudinal piezoelectric coefficient d_33_ reaching 10 pC/N.^113^ Similarly, Feng et al. utilized the induction effect of niobium carbide Mxene (Nb_2_CT_x_) nanosheets to facilitate the oriented growth of β-glycine within the nanoconfined fiber channels, forming nano-composite fibers with interface-locked arrangements and exhibiting excellent longitudinal and transverse piezoelectric output.^115^ Mechanical annealing refers to a process in which amino acid nucleation is induced by temperature variation, followed by drying and subsequent compression under a given pressure to obtain film-like crystals. For example, mechanical annealing of isoleucine crystal films increased d_33_ from 0.1 to 1.2 pC/N. Biodegradable sensors fabricated from this material degrade by 60% within six weeks and sensitively monitor physiological activities including muscle contraction and respiration.^116^

**Figure 5:**
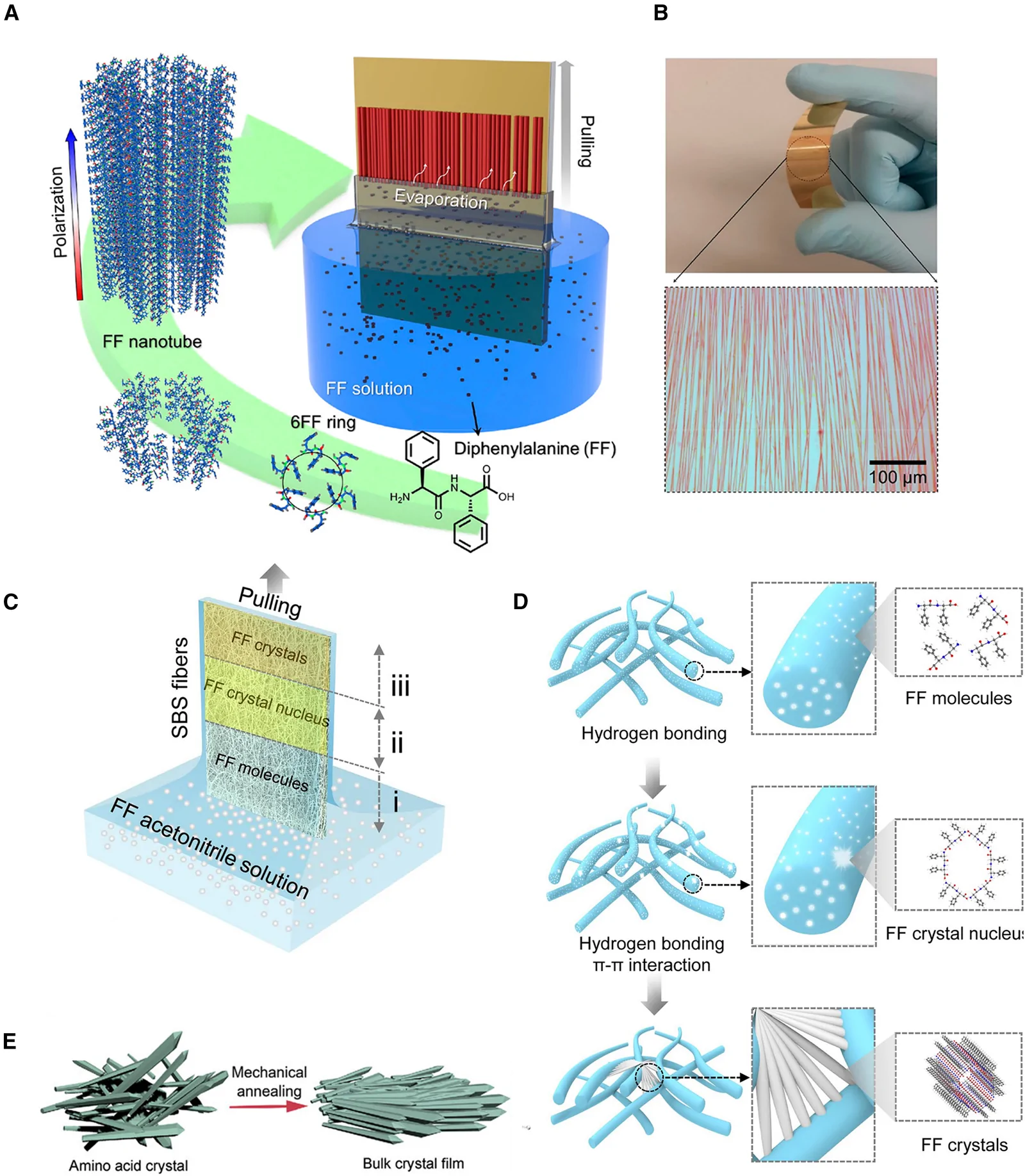
Mechanical force driven methods for fabricating piezoelectric biomaterials (A) Dip coating of horizontally aligned FF nanotubes and schematic of the meniscus force-driven dip-coating process. (B) Photograph of the FF nanotubes on a flexible substrate^112^ (adapted from Lee et al.^112^ with permission). (C) Synthesis and self-assembly mechanism of FF-CFs. Schematic illustration of the micro-permeation pulling process of SBS fibers and the self-assembly process of FF molecules. (D) SBS fibers are employed as a nanoconfinement carrier for the self-assembly of FF, yielding elastic FF crystal fibers featuring a distinctive Mortise-Tenon structure^113^ (Adapted from Ma et al.^113^ with permission). (E) Schematic of the mechanical annealing of isoleucine crystals. The alignment of the crystals is significantly enhanced after mechanical annealing.^6^

Overall, mechanically driven fabrication offers notable advantages in processing speed, scalability, operational simplicity, and design flexibility (Figure 5; Table 4).

**Table 4:** Driving forces and preparation techniques for enhancing piezoelectricity in amino acids and peptides

Driving forces	Preparation techniques	Principle	Examples	Reference
Electric field	electrohydrodynamic spray method	high voltage is applied to a flowing liquid, converting it into microscopic vaporized particles, which are deposited as an oriented layer.	β-glycine film *d*_33_ = 11.2 pm/V	Zhang et al.^77^
	corona discharge method	utilizes a high-voltage electric field to align the dipoles within the liquid, thereby polarizing the material.	PBLG-PMMA composite film *d*_33_ = 23 pC/N	Farrar et al.^82^; Huang et al.^111^
	electrospinning	utilizes a strong electric field to subject the polymer jet to electrostatic stretching and mechanical traction, promoting *in-situ* polarization of dipoles in the polymer chains.	PBLG fiber *d*_33_ = 25 pC/N	Farrar et al.^80^; Wang et al.^105^
Magnetic field	/	applying an oriented magnetic field induces the oriented alignment and polarization of biocrystals, generating piezoelectricity.	Co-assembled diphenylalanine derivative and Fe_3_O_4_ form nanorods with a piezoelectric coefficient of 121.9 pm/V	Pandey et al.^103^; Huang et al.^111^
Mechanical force	meniscus	utilizing the meniscus formed at the solid-liquid-gas triple phase interface, solute molecules are induced to arrange in an ordered manner via capillary flow during substrate pulling.	FF nanotubes *d*_15_ = 46.6 pm/V	Lee et al.^114^
	nanoconfined self-assembly	confining molecules within nanoscale spaces to enable their directed self-assembly in a restricted environment.	FF-CFs mortise-tenon structure *d*_33_ = 10 pm/V	Ma et al.^113^
	mechanical annealing and stress-induced recrystallization	mechanical annealing exploits temperature-induced nucleation followed by compressive pressing to convert amino acid aggregates into crystalline thin films.	L-isoleucine crystal film *d*_33_ = 1.2 pC/N	Cheng et al.^116^

Under different external-field-assisted fabrication technologies, the self-assembled structures and properties of amino acids exhibit significant differences. Taking FF as an example, FF monomers readily self-assemble into nano/microtubes in solution. However, in their natural state, these self-assembled FF tubes are macroscopically randomly arranged, leading to the cancellation of their overall dipole moments and resulting in near-zero macroscopic piezoelectricity. Therefore, introducing an external field to control their orientation is an important method for obtaining macroscopically high-performance FFs (Table 5).

**Table 5:** Different external-field-assisted fabrication technologies apply to the respective characteristics of FF

Driving force	Types	Molecular morphology	Alignment Direction/Piezoelectric coefficient	Advantages	Disadvantages	Reference
Electric Field	electric field-assisted self-assembly	vertical FF peptide microrods with controlled inherent polarization	along the electrical field direction (*d*_33_ = 17.9 pm/V)	(1) unidirectional polarization alignment (2) simple equipment	(1) challenging to scale (2) environmentally sensitive (temperature and humidity of the self-assembly environment)	Nguyen et al.^86^
Magnetic Field	dual-scale polarization	the majority of nanorods responded to the magnetic field and oriented in the same direction	along the magnetic field (elevates the d_eff_ from 10.4 to 121.9 pm/V)	(1) uniform Alignment (2) reduced magnetic field requirements (3) high cost-effectiveness (4) highly scalable production capabilities	(1) typically requires high magnetic field strength. (2) large-scale, highly uniform macroscopic orientation, with high requirements for equipment	Huang et al.^117^
Mechanical Force	nanoconfinement self-assembly	mortise-tenon structure	in-plane (IP) alignment (*d*_33_ = 10 pC/N)	(1) elasticity, flexibility, piezoelectricity, breathability, and physical stability. (2) highly scalable production capabilities	high purity requirements for raw materials	Ma et al.^113^

## Applications of piezoelectric amino acids and peptides in biomedical fields

### Biosensing and piezoelectric nanogenerators

Piezoelectric amino acids and peptides are well-suited for biosensors and piezoelectric nanogenerators. Biosensors convert physiological and biochemical signals from living organisms into quantifiable electrical outputs. Physiological activities such as breathing, heartbeat, and muscle contraction generate physiological electrical signals that are typically too weak to be easily detected. Sensors are capable of amplifying weak signals and efficiently acquiring reliable physiological data, with applications ranging from patient monitoring and rehabilitation assessment to detection of abnormal motor behavior in neurological conditions such as Parkinson’s disease. Sensors fabricated from piezoelectric amino acids and peptides can harvest ambient mechanical energy while demonstrating excellent biodegradability and biocompatibility.^96^ Piezoelectric nanogenerators exploit mechanical energy from the physiological environment, aligning material dipoles to generate a potential difference and thereby converting mechanical energy into electrical energy, thus providing sustainable power for implantable medical devices.^118^

#### Capturing and monitoring of physiological electrical signals

Implantable or wearable piezoelectric sensors can amplify weak physiological electrical signals for continuous monitoring. For example, a biodegradable force sensor fabricated from fluorinated Z-Phe was implanted subcutaneously into the rat chest, recording a peak-to-peak voltage (Vpp) of approximately 30 mV at a respiratory rate of 60–70 breaths per minute.^37^ Similarly, a composite film of PLA-encapsulated glycine and PVA implanted subcutaneously into rat hindlimbs generated Vpp reaching 100–150 mV during leg extension. The device degraded completely within one day of implantation without inducing inflammatory responses, demonstrating both biocompatibility and degradability.^96^ Furthermore, an elastic FF-CFs sensing system, fabricated via nanoconfinement-directed self-assembly, can conformally adhere to human skin and enable high-fidelity detection of both hand tremors characteristic of Parkinson’s disease and subtle internal movements such as heartbeat, breathing, and diaphragm motion.^113^

For sensors targeting more easily identifiable limb movements, the design priority shifts to highly sensitive dynamic signal detection for real-time capture. A biodegradable isoleucine film force sensor offers sensitive dynamic responses across the 0.5–10 Hz range, with output voltage increasing from 25 mV to 100 mV as the finger flexion angle increases. It can stably resolve subtle wrist flexion and gentle gestures such as swallowing, making it applicable to daily activity monitoring and rehabilitation evaluation.^116^ A piezoelectric sensor based on Cu^2+^ and glutamic coordination assemblies generates an open-circuit voltage exceeding 3.0 V under an applied force of 55 N. It can effectively capture the force variations in the cervical and lumbar spine during daily activities, facilitating real-time monitoring of spinal health.^97^ A glycine film pressure sensor developed by Tan et al. can discriminate four distinct occlusal contact patterns, providing a novel visualization tool for denture evaluation and orthodontic treatment.^119^

#### Piezoelectric nanogenerators

The relatively high piezoelectric coefficients of FF peptides make them attractive for nanogenerator applications.^120^ Electric field-guided polarization alignment of FF dipoles enables vertical orientation of the nanotubes. Under an external pressure of 60 N, the resulting device achieves an open-circuit voltage (V_OC_) of 1.4 V, and a short-circuit current (I_SC_) of 39.2 nA.^83^^,^^114^ Under a mechanical force of 31 N, L-tyrosine crystal film exhibits *V*_oc_ = 0.5 V and *I*_sc_ = 35 nA.^121^ Yang et al. implanted a biodegradable glycine and PVP film subcutaneously in pigs; upon ultrasound activation, it generated an electrical output of approximately 3.6 V and 10 μA, which is sufficient to charge small implantable devices such as pacemakers and defibrillators.^122^

These biodegradable piezoelectric sensors and generators represent a promising renewable and biocompatible power source for next-generation implantable electronics.

### Tissue regeneration and repair

#### Skin wound healing

Traditional piezoelectric materials such as PZT can promote cell proliferation and tissue regeneration in wound healing. However, they pose risks of cytotoxic element release and, owing to their mechanical rigidity, are poorly suited to the contoured geometry of skin surfaces, limiting their clinical translation.^21^^,^^23^^,^^123^ Amino acid- and peptide-based biodegradable piezoelectric materials not only facilitate wound healing but are also fully resorbable by the body, eliminating the need for secondary surgical intervention.^124^

Su et al. prepared a glycine and PVA composite film by solution casting that generated a 6 V voltage output under 25 N force. The material enhanced collagen deposition, reduced inflammatory responses in rat skin defect models, and increased wound closure rate by 20% on day 7 post-implantation.^125^ Wu et al. developed a γ-glycine and PVA composite film that, upon ultrasound stimulation, effectively promotes collagen deposition, angiogenesis, and anti-inflammatory responses while exhibiting good biocompatibility.^126^ A β-glycine and chitosan composite generates a voltage upon mechanical deformation, guiding cell migration and accelerating wound closure while exhibiting excellent biodegradability.^127^

#### Nerve regeneration

Nerve impulse conduction relies on electrical signal propagation. Piezoelectric materials can enhance electrical conductivity in damaged nerve tissues and thereby promote nerve regeneration. Li et al. synthesized polycaprolactone-β-glycine composite nanofibers and implanted them into rat sciatic nerve defect models. The mechanical vibration-induced piezoelectric stimulation effectively promoted myelination and neurite outgrowth in Schwann cells, ultimately achieving functional repair of long-gap nerve defects with approximately 99% motor recovery and 96% nerve conduction recovery.^128^

#### Bone regeneration

Bone is inherently a natural piezoelectric tissue. The collagen fibers that constitute the bone matrix possess a unique triple-helix structure and exhibit a transverse piezoelectric effect along the fiber axis.^17^^,^^18^^,^^129^ Mechanical loading of bone during movement converts mechanical stimuli into electrical signals. These signals regulate osteoblast proliferation and differentiation by modulating mechanosensitive ion channels such as Piezo1 and associated downstream signaling pathways. Piezoelectric biomaterials can provide a bioelectric field to damaged bone tissue, promoting bone growth and remodeling.^130^

Glycine possesses intrinsic anti-inflammatory properties and enhances bone mineral density.^131^^,^^132^ Electrospun composite scaffolds incorporating glycine with biocompatible polymers such as poly(L-lactic acid) (PLLA) and PVA hold considerable promise for bone tissue engineering.^32^ Ultrasound-driven piezoelectric β-glycine and PLLA composite piezoelectric films significantly promoted bone regeneration in a mouse humeral fracture model, outperforming pure PLLA controls.^133^ FF-based peptide materials also play a crucial role in bone repair. The voltage output of piezoelectric scaffolds influences the differentiation fate of mesenchymal stem cells (MSCs); lower voltage outputs (approximately 25 mV) favor chondrogenic differentiation, while higher outputs (40–80 mV) promote osteogenic differentiation. The FF vertically aligned microrod array (*d*_33_ = 18 pC/N) is well-suited to drive this voltage-dependent differentiation.^134^^,^^135^ Zhang et al. designed an FF-modified piezoelectric decellularized cartilage extracellular matrix and a piezoelectric-conductive gelatin hydrogel scaffold. *In vivo* experiments in a porcine joint defect model demonstrated that the upper scaffold layer generated a positive charge to promote articular cartilage repair, while the lower layer generated a negative charge to promote osteogenesis.^136^ Fmoc-modified FF can self-assemble into supramolecular hydrogels. The dried nanofibrils exhibit a non-centrosymmetric β-sheet structure with piezoelectric properties and tissue-matched mechanical compliance.^84^^,^^137^ Incorporation of cell-adhesive arginine-glycine-aspartate motifs into the piezoelectric Fmoc-FF hydrogel enhances the proliferation and survival of MSCs while promoting bone repair.^130^^,^^137^

Biodegradable piezoelectric materials based on amino acids and peptides promote cell proliferation, tissue regeneration, and functional recovery through piezoelectric stimulation in applications such as skin wound healing, nerve regeneration, and bone repair, while circumventing the toxicity, mechanical mismatch, and need for secondary surgery that limit conventional piezoelectric materials.

### Antimicrobial

Antimicrobial peptides possess broad-spectrum antimicrobial activity. To enhance their antimicrobial efficacy, Tan et al. integrated piezoelectric functionality with antimicrobial peptide design. They engineered the peptide FFRKSKEK (FFRK8) based on the core fragment of the human antimicrobial peptide LL-37 and incorporating the FF piezoelectric unit. Upon ultrasonic activation, this peptide achieves a piezoelectric coefficient d_33_ as high as 66.55 pm/V. Molecular dynamics simulations indicate that ultrasound enhances membrane penetration by FFRK8. Meanwhile, the FF fragment simultaneously releases free electrons via the piezoelectric effect, generating reactive oxygen species (ROS) that disrupt bacterial electron transport chains and eliminate multidrug-resistant pathogens. In a goat model of refractory intervertebral disc infection, FFRK8 demonstrated superior therapeutic efficacy compared to vancomycin, underscoring the clinical potential of ultrasound-activated piezoelectric antimicrobial peptides for combating antibiotic-resistant infections.^71^

### Cancer treatment

**Figure 6:**
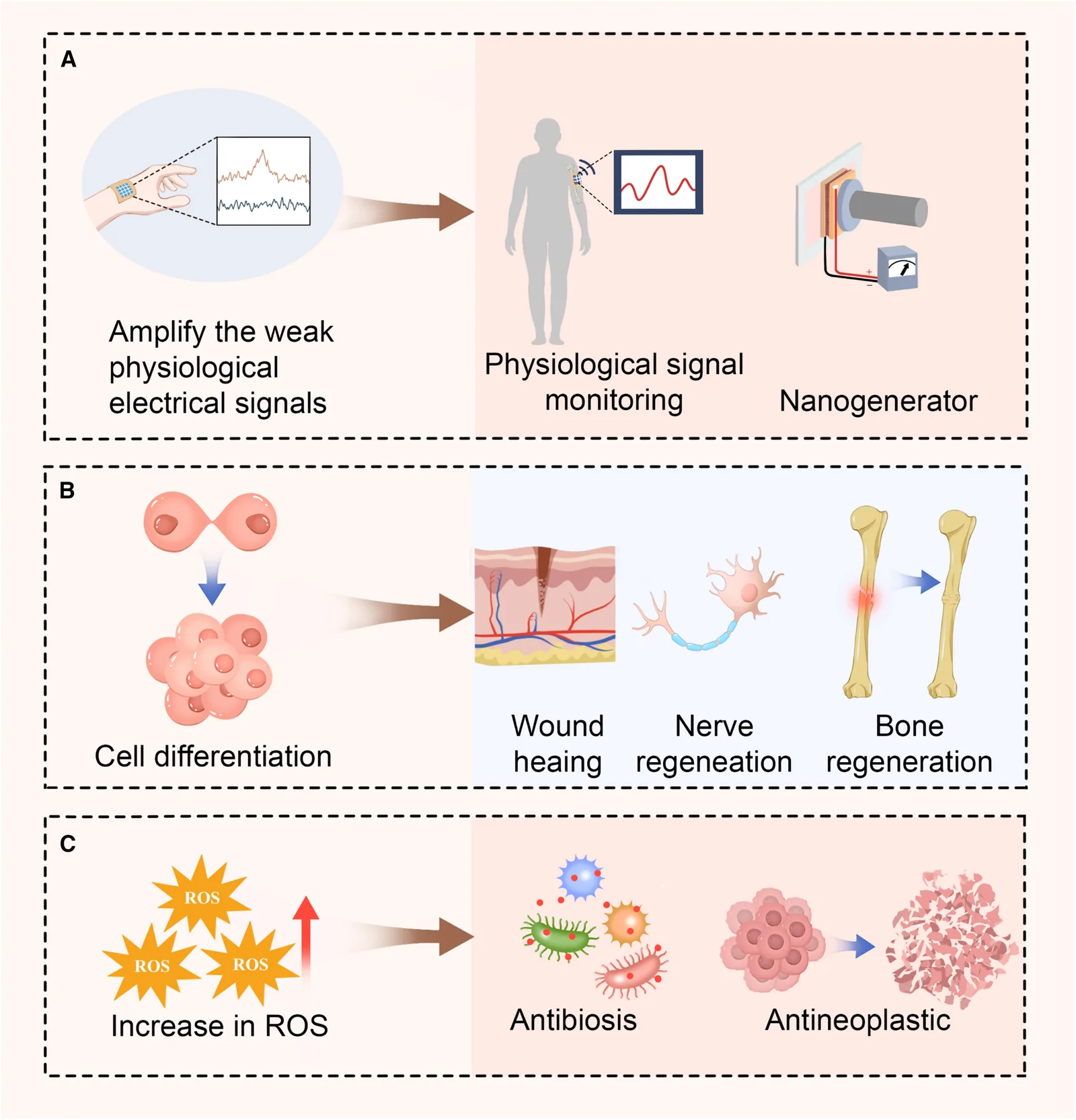
Multifunctional applications of piezoelectric amino acids and peptides in biomedicine (A) Biosensing and nanogeneration. (B) Skin wound healing, nerve regeneration, and bone growth. (C) Antibacterial and antitumor applications.

Piezoelectric amino acids and peptides offer advantages, including non-invasiveness, low energy dissipation, and deep tissue penetration, making them suitable for the treatment of deep-seated tumors. Their primary mechanisms include direct ROS-mediated tumor cell killing and drug delivery carrier functions that synergize with chemotherapy. Regarding direct cytotoxic mechanisms, ultrasound-induced piezoelectric activation of nanotubes self-assembled from the modified dipeptide phenylalanine-αβ-dehydroxyphenylalanine elevated intracellular Ca^2+^ concentrations in C6 glioma cells. This augments ROS production and disrupts glioma cell metabolism. Loading doxorubicin into these nanotubes further enhanced anticancer efficacy through synergistic piezoelectric-chemotherapeutic action.^138^ Chorsi et al. implanted glycine and polycaprolactone composite nanofibers into an orthotopic glioblastoma mouse model. Ultrasound stimulation reversibly opened the blood-brain barrier, facilitating the targeted delivery of chemotherapeutic agents and doubling mouse survival time relative to controls.^139^ Piezoelectric amino acids and peptides enable non-invasive treatment of deep-seated tumors via ultrasound-induced ROS generation and reversible blood-brain barrier opening, while serving as drug delivery carriers to achieve synergistic chemo-piezoelectric therapy (Figure 6).

## Conclusions and perspectives

Piezoelectric materials primarily convert mechanical energy into electrical energy due to structural or molecular asymmetry, causing a change in dipole moment when subjected to force. Conventional piezoelectric ceramics and PVDF films are limited by their non-degradability and suboptimal biocompatibility, restricting their applicability in implantable devices. Piezoelectric amino acids or peptides constitute an emerging class of bio-piezoelectric materials that combine biodegradability with high biocompatibility. Since the intrinsic piezoelectric coefficients of unmodified natural amino acids and peptides are modest, various enhancement strategies have been developed: chemical modifications including fluorination and acetylation, composite formulations with polymers, and dopant incorporation all serve to improve piezoelectric output.^89^^,^^90^^,^^96^^,^^98^ The application of electric, magnetic, or mechanical fields can induce molecular polarization along the field direction, achieving unidirectional dipole alignment and substantially enhancing piezoelectric performance.^103^^,^^111^^,^^114^ Piezoelectric amino acids and peptides obtained through these fabrication methods demonstrate broad application prospects in biomedical applications. In biosensing and energy harvesting, composite materials based on FF and other amino acids can sensitively detect weak physiological signals, including respiration, heartbeat, and muscle contraction, and then convert biomechanical energy into bioelectrical energy to provide sustainable power for implantable devices.^122^ In tissue regeneration, they can be used for skin, nerve, and bone regeneration. For instance, voltage-dependent control of MSC differentiation enables targeted chondrogenic or osteogenic induction, with promising results in preclinical joint defect models.^134^^,^^135^ In antibacterial therapy, the integration of piezoelectric FF sequences within antimicrobial peptides, combined with ultrasonic actuation, enables highly efficient elimination of drug-resistant bacteria.^71^ In oncology, piezoelectric amino acid assemblies can generate ROS upon ultrasound stimulation to directly kill tumor cells and facilitate drug delivery.^138^

Nevertheless, several challenges must be addressed before clinical translation can be realized. First, when the applied force is aligned with the polarization direction, the mechanical energy is most efficiently transduced into changes in dipole moments, yielding the maximum polarization charge in the longitudinal mode. Consequently, the longitudinal piezoelectric coefficient d_33_ is one of the most critical performance metrics for most piezoelectric applications. Compared with traditional inorganic piezoelectric ceramics, the longitudinal piezoelectric coefficients of amino acid and peptide materials are generally lower. When polarized charges are subjected to axial pressure, they are prone to charge self-cancellation due to internal lattice relaxation. Additionally, chiral amino acids serve as the molecular basis for piezoelectricity; however, it is the non-centrosymmetric macroscopic arrangement that constitutes the direct prerequisite for macroscopic piezoelectricity. Peptides tend to self-assemble into antiparallel structures that have lower free energy, causing the molecular dipole moments to cancel each other out, resulting in a macroscopic d_33_ value approaching zero. Many peptides, such as α-helical peptides, inherently possess strong one-dimensional axial symmetry, which gives rise to a high shear piezoelectric constant d_14_ but a low longitudinal piezoelectric constant d_33_ in the out-of-plane direction. Therefore, amino acids can be modified at the side chains, or strong electronegative atoms, such as fluorine, can be introduced to enhance the local dipole moments of the molecules. Alternatively, some physical control methods can be employed to achieve uniform polarization or uniaxial orientation of the piezoelectric biomaterials, enhancing the macroscopic d_33_ output. Second, the precise regulation of piezoelectric properties requires further investigation. There is a need to establish quantitative structure-property relationships linking molecular structure, dipole moment, and piezoelectric response to enable predictable performance optimization from the molecular level to the macroscopic scale. As an illustrative example, fluorination of Z-Phe increases the molecular dipole moment from 3.19 D to 4.42 D, yielding a piezoelectric coefficient of 50.36 pm/V, approximately one order of magnitude higher than that of the unmodified amino acid.^16^ Third, due to abundant proteases and peptidases in the human body, the polypeptide backbone, which contains easily hydrolyzable peptide bonds, is prone to rapid degradation *in vivo*. The *in vivo* degradation time of unmodified piezoelectric amino acid materials is typically only 1–2 weeks. Therefore, we can use hydrophobic flexible polymers for encapsulation of peptides to provide a physical barrier. For example, PLA encapsulation of glycine/PVA composite films extends the *in vivo* degradation time from 1 day to 4 weeks.^96^ Alternatively, layered membrane encapsulation can isolate body fluids and reduce the *in vivo* degradation rate of piezoelectric amino acids and polypeptides, such as integrating the amino acid crystal films with conducting PAN electrodes and biodegradable coating layers to fabricate packaged force sensors, which can still monitor dynamic physiological activities at 4 weeks.^6^ Moreover, replacing natural L-amino acids with D-amino acids can significantly improve the metabolic stability of peptides.^140^ Finally, insufficient long-term biosafety validation is another key limitation for clinical translation. Although amino acids and peptides themselves are non-toxic, a rapid degradation of devices may lead to a sharp increase in local degradation products, such as phenylalanine, which could alter osmotic pressure or the local pH, provoking cellular stress. Existing *in vivo* studies have not observed overt inflammation or systemic toxicity, but systematic monitoring of the relationship between degradation kinetics and the local metabolic microenvironment is still necessary.

The field currently faces multiple limitations that are interconnected and mutually constraining. Chemical modifications can effectively enhance piezoelectric performance while potentially altering material degradability. Conversely, strategies aimed at regulating degradation behavior may affect biocompatibility. Therefore, future research should strive to achieve a dynamic balance among piezoelectric performance, degradation rate, and biosafety to promote the clinical application of piezoelectric amino acid and peptide materials.

## Acknowledgments

The authors thank the support of Northwest University of Xi’an, Shaanxi, China, as well as BioRender (biorender.com) for providing items for drawing scheme graphs. We thank the grants from the National Natural Science Foundation of China (32301088) and Scientific Research Program Funded by Shaanxi Provincial Education Department (Proram No, 23JK0688).

## Author contributions

M.Z.: writing the first draft and visualization (figure design); H.W.: investigation and visualization; H.L.: visualization (table preparation); D.Z.: investigation; T.M.: writing and reviewing the manuscript; J.W.: writing and reviewing the manuscript.

## Declaration of interests

The authors declare no conflict of interests.
